# A Left Atrial Myxoma Masquerading As Acute Coronary Syndrome

**DOI:** 10.7759/cureus.29300

**Published:** 2022-09-18

**Authors:** Subash Nepal, Martha L Caicedo Murillo, Kamala Ojha, Madhab Lamichhane

**Affiliations:** 1 Cardiovascular Disease, Upstate University Hospital, Syracuse, USA; 2 Pathology, Upstate University Hospital, Syracuse, USA; 3 Internal Medicine, Upstate University Hospital, Syracuse, USA; 4 Cardiology, Upstate University Hospital, Syracuse, USA

**Keywords:** echocardiogram, acute coronary syndrome, embolism, left atrium, cardiac myxoma

## Abstract

Acute coronary syndrome is caused by a rupture of atherosclerotic plaque with superimposed thrombus formation. Non-ST-segment elevation acute coronary syndrome (NSTE-ACS) occurs when occlusion of the epicardial coronary artery by a thrombus causes partial thickness myocardial ischemia and an ischemic cascade downstream. Cardiac masses are known to produce symptoms predominantly by local obstruction or systemic embolism. Coronary artery tumor embolism causing acute coronary syndrome is a rare presentation of cardiac mass. We report NSTE-ACS as the initial presentation of the left atrial myxoma in a 62-year-old woman. She presented to the emergency department with acute onset severe anginal chest pain, diaphoresis, and dizziness. Her serial electrocardiograms (ECGs) were normal, and serial troponins were elevated, consistent with non-ST-segment elevation acute coronary syndrome. Cardiac catheterization revealed insignificant coronary artery disease, and transthoracic and transesophageal echocardiograms showed a left atrial mass attached to the interatrial septum consistent with myxoma. The patient underwent surgical excision, and histopathology was consistent with myxoma. Her symptoms subsided after surgery. Primary cardiac tumors are very uncommon and can present with myriad symptoms, from tumor embolism, local cardiac effects, to constitutional symptoms. Although embolism to other organs has been reported, left atrial myxoma presenting as an acute coronary syndrome is very uncommon.

## Introduction

Primary cardiac neoplasms are very rare, with an incidence rate of about 0.2% at autopsy [1]. Cardiac tumors can be classified into benign, malignant (primary malignant, and secondary), and tumor-like masses (Lambl’s excrescences, papillary fibroelastoma, endocarditis, and blood cysts). About 90% of primary cardiac neoplasms are benign and arise from the myocardium, heart valves, and pericardium [2]. Studies have shown that secondary cardiac neoplasms are about 100 times more common than primary cardiac tumors [3]. Common primary benign cardiac tumors are myxoma, lipoma, papillary fibroelastoma, rhabdomyoma, fibroma, and cardiac paraganglioma [2]. Commonly encountered malignant cardiac tumors are angiosarcoma, leiomyosarcoma, rhabdomyosarcoma, osteosarcoma, undifferentiated sarcoma, primary cardiac lymphoma, and mesothelioma [2]. The tumors known to metastasize to the heart in order of decreasing frequency are primary lung cancer, breast cancer, hematological malignancy, pleural mesothelioma and, melanoma, ovarian and renal cancers [4]. Cardiac masses can have a myriad of clinical presentations, commonly present with local cardiac effects and constitutional/ systemic symptoms by releasing inflammatory cytokines and embolic phenomenon. Although pulmonary and systemic embolism with cardiac myxoma is common, coronary artery embolism is very rare. We describe a case of acute coronary syndrome as an atypical presentation of left atrial myxoma.

## Case presentation

**Figure 1:**
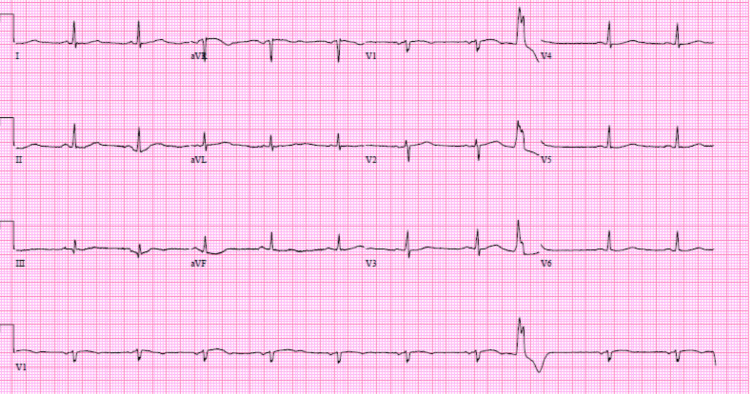
ECG on presentation

A 62-year-old woman with a past medical history of hyperlipidemia, hypothyroidism, a family history of coronary artery disease, and a current smoker presented to the emergency department with sudden onset of left-sided crushing chest pain. Chest pain was pressure-like, 9/10 in intensity, radiated to the left arm and jaw, and associated with diaphoresis. Physical examination revealed normal first and second heart sounds without any murmur or rales. An ECG showed premature ventricular and atrial complexes and was negative for ST-segment changes (Figure 1).

Serial high sensitive troponin T was elevated to 51 ng/L (at presentation) and 144 ng/L (in three hours with a delta of 93 ng/L). The patient was treated with a loading dose of clopidogrel 300 mg, aspirin 162 mg, sublingual nitroglycerin, and started on a heparin drip. Cardiac catheterization revealed insignificant coronary artery disease with Thrombolysis in Myocardial Infarction (TIMI) 3 flow (Videos 1-6).

**Video 1:** Coronary angiogram left anterior oblique caudal view showing a normal right coronary artery and its branches

**Video 2:** Coronary angiogram right anterior oblique caudal view showing a normal right coronary artery and its branches

**Video 3:** Coronary angiogram left anterior oblique caudal view showing a normal left main, proximal left anterior descending, and circumflex arteries and branches

**Video 4:** Coronary angiogram right anterior oblique caudal view showing a normal left main, left anterior descending, and circumflex arteries and branches

**Video 5:** Coronary angiogram right anterior oblique cranial view showing a normal left anterior descending artery with normal caliber diagonal branches

**Video 6:** Coronary angiogram left anterior oblique cranial view showing a normal left main, left anterior descending, and diagonal branches

**Figure 2:**
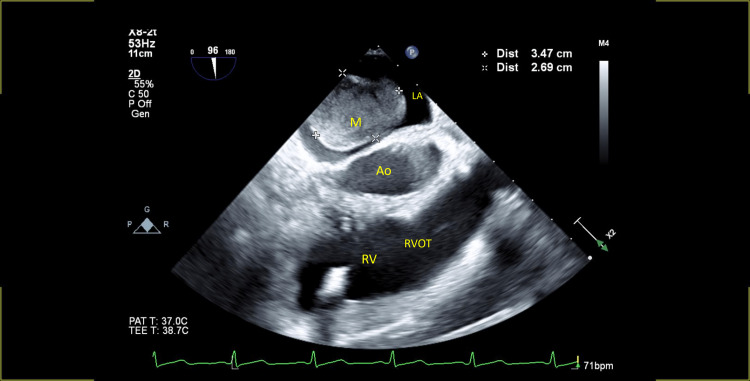
Transesophageal echocardiogram, mid-esophageal right ventricular inflow and outflow view showing an oval well-defined mass in the left atrium arising from the interatrial septum Ao - aortic root in short axis, LA - left atrium, M - mass, RV - right ventricle, RVOT - right ventricular outflow tract

Transthoracic echocardiogram showed a normal left ventricular systolic function, no regional wall motion abnormalities, and a large sessile heterogenous globular echodensity 2.9 x 2.6 cm in the left atrium attached to the interatrial septum suggestive of myxoma (Video 7-8, Figure 2).

**Video 7:** Transthoracic echocardiogram, parasternal long axis view showing a globular mass in the left atrium attached to the interatrial septum Ao - aortic root, LA - left atrium, LV - left ventricle, M - mass, RV - right ventricle

**Video 8:** Transthoracic echocardiography, apical four-chamber view showing a globular mass in the left atrium arising from the interatrial septum suggestive of myxoma Ao - aortic root, LA - left atrium, LV - left ventricle, M - mass, RA - right atrium, RV - right ventricle, MV - mitral valve

**Figure 3:**
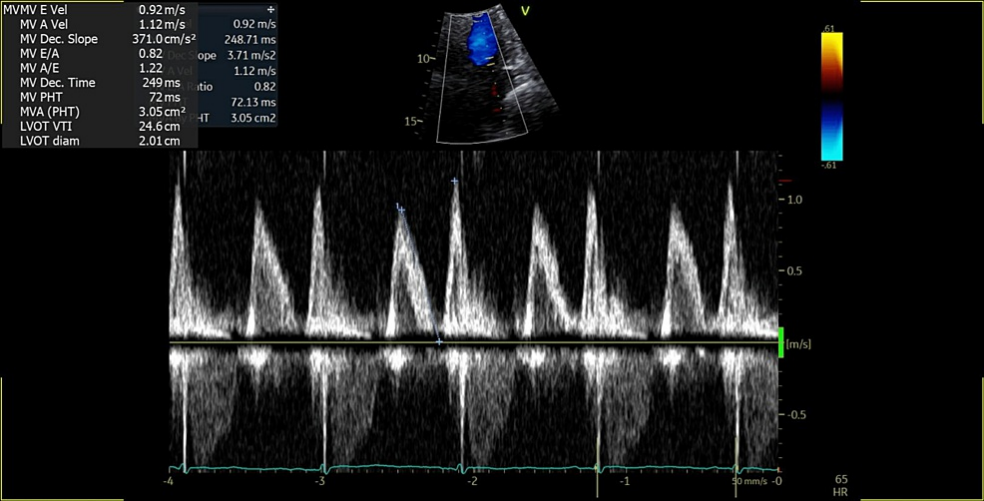
Pulse wave doppler across the mitral valve showing normal transmitral flow without any obstruction

The anatomic extent of the tumor was further studied by transesophageal echocardiography, which did not show the involvement of any other cardiac chambers. The mass was localized to the interatrial septum and did not cause mitral valve obstruction (Figure 3, Videos 9-11).

**Video 9:** Transesophageal echocardiography, mid-esophageal right ventricular inflow and outflow tract view showing a globular heteroechoic mass in the left atrium attached to the interatrial septum suggestive of myxoma AV - aortic valve, short axis, IAS - interatrial septum, LA - left atrium, M - mass, RA - right atrium, RV - right ventricle, RVOT - right ventricular outflow tract, TV - tricuspid valve

**Video 10:** Transesophageal echocardiogram, mid-esophageal four-chamber view showing a globular mass in the left atrium attached to the interatrial septum IAS - interatrial septum, LA - left atrium, LV - left ventricle, M - mass, MV - mitral valve, RA - right atrium, RV - right ventricle, TV - tricuspid valve

**Video 11:** Transesophageal echocardiography, midesophageal four-chamber view zoomed in the left atrium showing a globular, heteroechoic mass (M) arising from the interatrial septum IAS - interatrial septum

**Figure 4:**
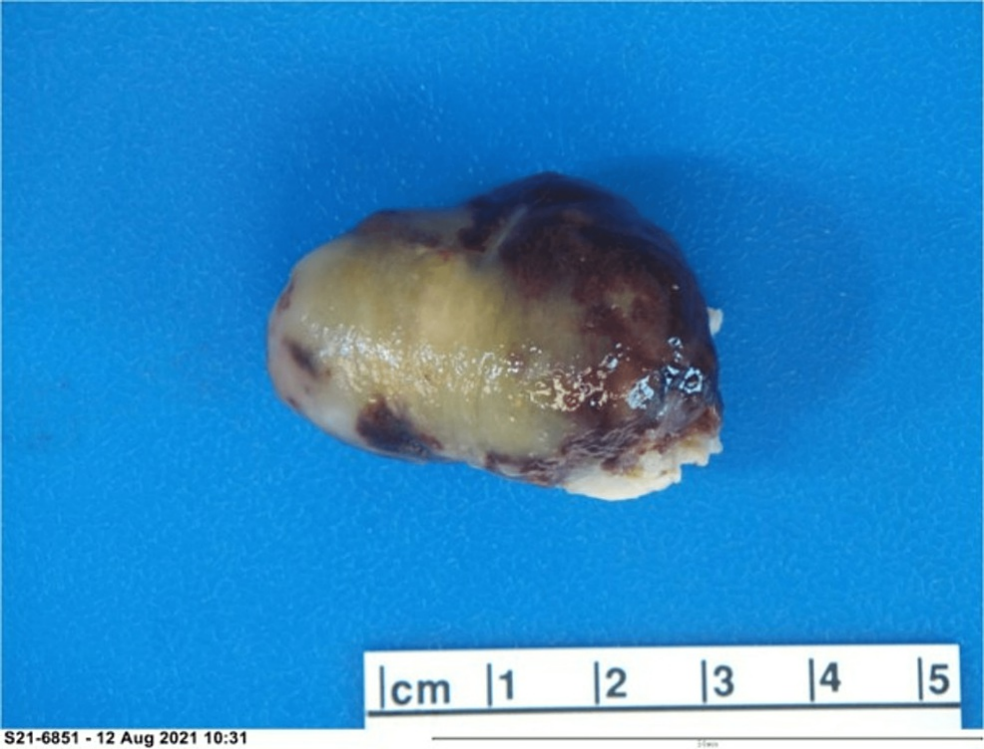
Gross specimen shows a 4.4 x 3.2 x 2.8 cm ovoid, yellow-red rubbery soft tissue mass. The surface was glistening and gelatinous.

**Figure 5:**
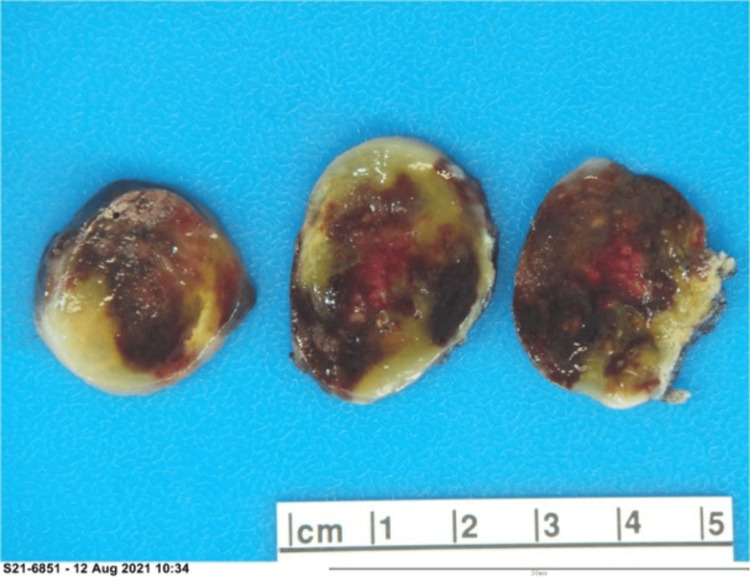
The cut surface reveals a yellow-red diffuse hemorrhagic and gelatinous appearance

Cardiac surgery was consulted and planned on surgical excision after a clopidogrel washout. Midline sternotomy and left atriotomy were performed under cardiopulmonary bypass. The sessile tumor measuring about 4.5 cm had the appearance of myxoma and was seen attached to the area of the fossa ovalis. The right atriotomy was performed, and the tumor was removed completely, along with a full-thickness piece of septum measuring 2 cm. The iatrogenic atrial septal defect was closed with sutures. The gross specimen showed an ovoid, well-defined, yellow-red soft tissue mass with diffuse hemorrhage and a gelatinous appearance (Figures 4-5).

**Figure 6:**
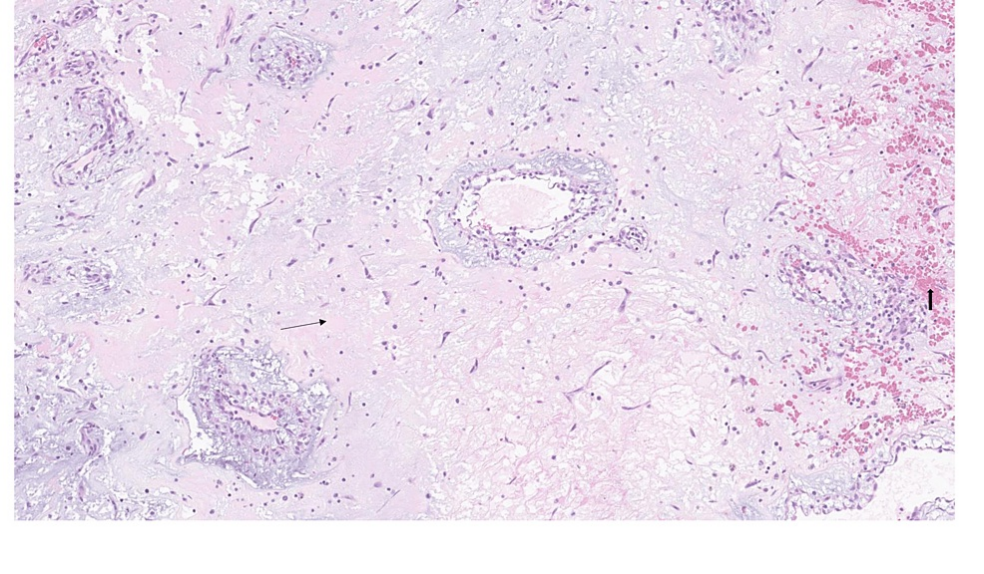
H&E section showing a paucicellular spindle neoplasm with a highly myxoid and vascularized stromal background, line arrow which is consistent with cardiac myxoma. Also, extravasated red blood cells (microhemorrhage), block arrows are common which is evident on the right side of the picture

**Figure 7:**
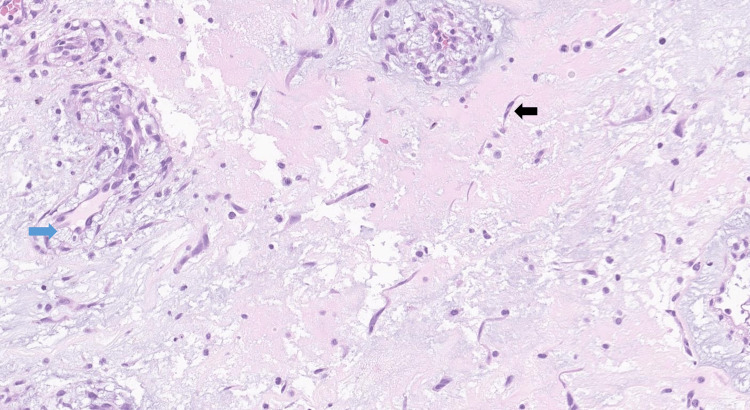
High power view of bland-looking spindle and stellate cells (black arrow), some arranged in perivascular spaces (blue arrow)

**Figure 8:**
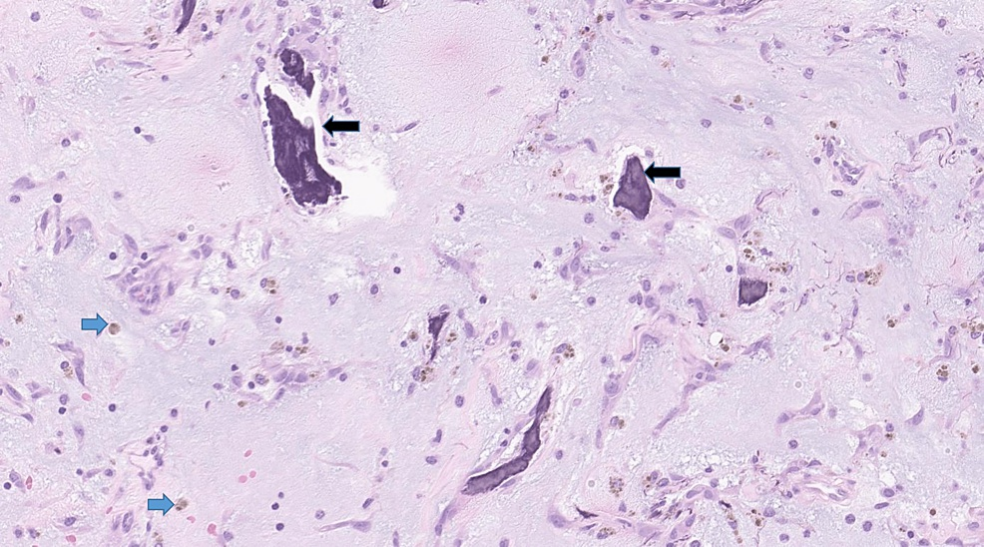
High magnification shows the Gamma-Gandy body (black arrow) which are dark-brown structures seen in the stroma of cardiac myxoma. These structures are the result of iron and calcium deposition on connective tissues (predominately collagen and elastin) and are often the result of intratumoral hemorrhage. Hemosiderin-laden macrophages are also seen as a result of bleeding (blue arrow)

Histology was consistent with myxoma (Figures 6-8).

She was discharged home in stable condition. Surveillance echocardiography after six months showed no recurrence.

## Discussion

The primary cardiac tumor is very rare, and myxoma is the most common primary cardiac tumor, constituting about 50% [5]. Most of them used to be diagnosed at autopsy in the past, but with the evolution of multimodality imaging, cardiac masses are being diagnosed more frequently now [2]. Myxoma arises from multipotent mesenchymal cells and is more common in women and in the third and sixth decades of life [1]. It may occur sporadically or as an autosomal dominant condition called Carney complex, where it presents in combination with other neoplasms like skin myxomas, cutaneous lentiginosis, pituitary neoplasm, testicular tumors, and primary adrenocortical tumors [6]. Myxomas typically arise from the interatrial septum adjacent to fossa ovalis. Valvular myxomas are very rare. It may rarely arise from the posterior and anterior atrial wall, left atrial appendage, left ventricle and right ventricle, and combined atrial and ventricular walls [1]. Multiple myxomas and myxomas with atypical locations are common in familial myxomas. Myxomas are commonly polypoid in gross appearance, often attached with a pedicle to the interatrial septum, and have spherical or oval shapes with smooth or bossellated surfaces [1]. The symptom triad commonly consists of systemic embolic manifestations, intracardiac obstructive symptoms like dyspnea on exertion, pulmonary edema, right heart failure, and constitutional symptoms like fever, weight loss, myalgia, and arthralgia. About 40% of patients with myxoma present with systemic embolism, and the central nervous system is involved in about 50% of them; coronary arteries involvement has been reported in only 0.06% [7, 8].

Our patient presented with non-ST-segment elevation myocardial infarction due to coronary artery embolism from left atrial myxoma. Physical examination commonly reveals a diastolic murmur in the mitral area due to left ventricular inflow obstruction. Late diastolic tumor “plop’’ occurs after the second heart sound when the tumor protrudes into the left ventricle. Our patient did not have any murmur as myxoma was sessile and localized to the interatrial septum only. Two-dimensional transthoracic echocardiography is diagnostic and has a sensitivity of about 95%. Transesophageal echocardiography has a sensitivity of about 95-100%, can detect tumors as small as 1-3 mm, and further demonstrate the site of attachment, mobility, concurrent involvement of the other chambers, and typical gross pathologic features like calcification, necrotic foci, and intratumor hemorrhage. Multimodality imaging like cardiac computed tomography scan (CT) and magnetic resonance imaging (CMR) can be used to evaluate extracardiac involvement and evaluate tissue components [1]. The tumor is benign and most of the time diagnosed by transthoracic and transesophageal echocardiography only, and multimodality imaging is not pursued for a typical myxoma with characteristic echocardiographic appearance and location, like in our patient. Surgery with sternotomy and atriotomy with resection of the tumor is curative with an excellent long-term prognosis. The recurrence rate of myxoma ranges from 5-14% [9]. Careful and complete resection should be performed as intraoperative fragmentation of the mass can cause embolism, and incomplete resection may lead to a recurrence [10]. Semi-annual surveillance echocardiography is recommended for four years after surgery to assess for recurrence [7]. Apart from embolism, the other rare causes of myocardial infarction with angiographically normal coronary arteries (MINCA) are coronary vasospasm, vasculitis, severe aortic stenosis, and hypertrophic obstructive cardiomyopathy [11-13]. 

## Conclusions

Cardiac tumors can have myriad presentations and left atrial myxoma can rarely present with acute coronary syndrome due to tumor embolism. An embolism from a cardiac tumor should always be suspected as one of the differential diagnoses of myocardial infarction with normal coronary arteries (MINCA). Transthoracic and transesophageal echocardiograms are diagnostic in most cases, and multimodality imaging is not required in a typical and localized myxoma. Surgical resection is curative with an excellent long-term prognosis. Follow-up surveillance echocardiography is recommended to assess for recurrence.
